# Retraction Note: Overexpression of TGFβ1 in murine mesenchymal stem cells improves lung inflammation by impacting the Th17/Treg balance in LPS-induced ARDS mice

**DOI:** 10.1186/s13287-022-02779-2

**Published:** 2022-03-01

**Authors:** Jianxiao Chen, Xiwen Zhang, Jianfeng Xie, Ming Xue, Ling Liu, Yi Yang, Haibo Qiu

**Affiliations:** Jiangsu Provincial Key Laboratory of Critical Care Medicine, Department of Critical Care Medicine, School of Medicine, Zhongda Hospital, Southeast University, 87 Dingjiaqiao Road, Nanjing, 210009 People’s Republic of China

## Correction to: Stem Cell Research & Therapy (2020) 11:311 10.1186/s13287-020-01826-0

The Editors-in-Chief have retracted this article. After publication, concerns were raised regarding potential image duplication, specifically:

- Several panels in Figs. 1a and 2b have been previously published in [1].
- The same actin western blot bands are presented (rotated 180 degrees) in Figs. 1E and 7D.
- Actin western blot bands appear highly similar in Fig. 6A 7d and 6B 3d.

Additionally, substantial text overlap was identified with the authors' earlier article [2].

The Editors-in-Chief therefore no longer have confidence in the presented data and the originality of the work.

Authors Jianxiao Chen, Jianfeng Xie, Ming Xue, Ling Liu and Yi Yang agree to this retraction. Authors Xiwen Zhang and Haibo Qiu have not responded to any correspondence from the editor or publisher about this retraction.
